# Ultrasound imaging of splenomegaly as a proxy to monitor colon tumor development in *Apc* ^min716/+^ mice

**DOI:** 10.1002/cam4.842

**Published:** 2016-08-03

**Authors:** Andrea Hodgson, Eric M. Wier, Kai Fu, Xin Sun, Fengyi Wan

**Affiliations:** ^1^ Department of Biochemistry and Molecular BiologyBloomberg School of Public HealthJohns Hopkins UniversityBaltimoreMaryland21025; ^2^ Department of Oncology and Sidney Kimmel Comprehensive Cancer CenterJohns Hopkins UniversityBaltimoreMaryland21287

**Keywords:** Colon tumor, in vivo imaging, splenomegaly, ultrasound

## Abstract

Animal models of colon cancer are widely used to understand the molecular mechanisms and pathogenesis of the disease. These animal models require a substantial investment of time and traditionally necessitate the killing of the animal to measure the tumor progression. Several in vivo imaging techniques are being used in both human clinics and preclinical studies, albeit at high cost and requiring particular expertise. Here, we report that the progression of splenomegaly coincides with and positively correlates to colon tumor development in *Apc* ^min716/+^ mice expressing a mutant gene encoding an adenomatous polyposis coli protein truncated at amino acid 716. Ultrasound image‐based spleen size measurement precisely mirrors splenomegaly development in vivo in the tumor‐laden *Apc* ^min716/+^ mice. Moreover, the spleen dimensions extracted from the ultrasound sonograms are positively correlated with normalized spleen weight and the number and area of colon tumors. Hence, we propose measuring the spleen size in vivo by ultrasound imaging as a novel approach to estimate splenomegaly development and to indirectly monitor colon tumor development in *Apc* ^min716/+^ mice. The widespread use of ultrasound machines in the laboratory setting, coupled with the fact that it is a noninvasive method, make it a straightforward and useful tool for monitoring the experimental progress of colon cancer in mice and determining end points without killing animals strictly for diagnostics purposes.

## Introduction

Colon cancer is among the most prevalent forms of cancer worldwide and a major cause of cancer death 1, 2, 3, 4, 5, 6. There are several animal models that have been developed to study colon cancer 7, 8, 9, 10, 11, 12, 13, 14, of which the *Apc* ^Min/+^ mouse model of Familial Adenomatous Polyposis (FAP) is similar in phenotype to the human disease 12, 13. Carrying a truncated allele of the adenomatous polyposis coli (*APC*) gene, cells within these mice are prone to spontaneously lose the remaining full‐length copy of the oncogenic *APC* gene, leading to tumor formation. APC regulates the levels and activity of *β*‐catenin within cells, and upon losing both functional copies of *APC* gene, the cell experiences constitutive activation of *β*‐catenin. The following dysregulation of Wnt signaling leads to the expression of proliferative genes 12, 15, 16 and genetically predisposes mice to tumor formation, particularly within the intestinal tract. Along with the accumulation of intestinal and colon tumors, *Apc* ^Min/+^ mice also develop splenomegaly of yet unknown origin 17, 18, 19. That said, the correlation between splenomegaly and colon tumor development has not been investigated previously. Moreover, splenomegaly has been linked to anemia, which is also detected in these animals; yet, no direct causation has been uncovered.

Monitoring tumor formation in humans relies on the use of scanning technologies including Positron Emission Tomography (PET), X‐ray Computed Tomography (X‐ray CT), Magnetic Resonance Imaging (MRI), and others, most of which have been adapted for use in animal research of disease 4, 14, 20, 21, 22, 23, 24, 25, 26, 27. These procedures are useful in that they provide direct evidence of tumor growth in localized areas albeit costly to do and not widely available. The development of confocal imaging techniques in vivo is advancing the field of colon cancer research for those who have access to the technology providing detailed images of the tissue in question 24. However, the prohibitive cost of obtaining these instruments and setting up these facilities provides to be a significant barrier to those who are interested in examining the general progression of disease in animal models. Furthermore, the injection of dyes, radiolabels, or lentivirus may have subsequent effects on the tumor microenvironment that could result in artifacts if used repeatedly. Alternatively, ultrasound imaging is a noninvasive procedure that can be performed with little manipulation of the subject and provide information regarding the tumor status 28, 29, 30, 31 and is a well‐established and widely‐used imaging technique 28, 32. In this report, we demonstrate that ultrasound imaging of the spleen can be used as a marker to monitor systemic tumor status within the colons of *Apc* ^min716/+^ mice that express a mutant gene encoding an adenomatous polyposis coli protein truncated at amino acid 716 33, 34. We examined the spleen size and colonic tumor number and load of animals of varying ages and determined that splenomegaly is positively correlated with the tumor development. In this way, we propose that measuring the spleen size in vivo can be utilized as a proxy to determine the degree of tumor formation in *Apc* ^min716/+^ mice, providing researchers the ability to more easily and noninvasively monitor the colon tumor development in vivo.

## Material and Methods

### Ethics statement and mice

All animal experiments were performed in accordance with Johns Hopkins University's Animal Care and Use Committee under the protocol MO13H349 and in direct accordance with the NIH guidelines for housing and care of laboratory animals. *Apc* ^min716/+^ mice 33, 34 were bred using an *Apc* ^min716/+^ male with wild‐type (*Apc* ^+/+^) females. Mice were maintained in a specific pathogen‐free facility and fed autoclaved food and water ad libitum.

### Colon adenoma staining and quantification

Colon adenoma staining and quantification were performed as previously described 33. Briefly, at the indicated time points, colons were resected under aseptic conditions from indicated mice, washed with ice‐cold PBS, and opened longitudinally. Colon tissues were mounted onto Histoplast PE (Thermo Scientific, Waltham, MA) and fixed in 10% neutral‐buffered formalin (3.7% formaldehyde, 1.2% methanol, 6.5 g/L sodium phosphate dibasic, 4.0 g/L sodium phosphate monobasic) at 25°C overnight, then washed in PBS, and stained with 0.2% (w/v) methylene blue (Sigma‐Aldrich, St. Louis, MO) for 1 h. Tissues were destained with multiple washes with PBS and destaining buffer (30% methanol and 10% acetic acid). Adenomas and aberrant crypt foci, identified as dark blue foci, in the cecum and the colon were counted using a dissection microscope. The tumor number and tumor load (the sum of the size of all tumors) present within the colon in a given mouse were determined as previously described 35.

### Spleen weight measurement

At the indicated time points, the body weight of indicated mice was determined prior to killing. After killing the mice, the spleen was gently excised under aseptic conditions, and spleen weight was measured by an analytical balance and normalized to body weight of individual mouse.

### Ultrasound imaging of the spleen in vivo and measurement of spleen dimensions

Mice spleens were imaged by ultrasound using a Vevo 770^®^ High‐Resolution Imaging System (VisualSonics, Toronto, Canada) following the manufacturer's instructions. Briefly, all fur was removed from the abdomen of mice using depilatory cream (Nair) and skin was washed with water to remove excess lotion. Mice were maintained anesthetized for the duration of the imaging and a heat lap was kept over the mice at all times to preserve body temperature. With a generous amount of warmed ultrasound gel applied to the skin, the mouse was restrained on the heated imaging platform with surgical tape and ultrasound images were taken of its abdomen using the RMV 704 scanhead. The sonograms were analyzed and the diameter, both along the shortest part as well as the widest part of the spleen, and the area was determined using ImageJ software (National Institutes of Health, Bethesda, MD).

### Statistical analyses

All statistical analysis was performed using GraphPad Prism version 6.0 (GraphPad Software, La Jolla, CA). Standard errors of means (S.E.M.) were plotted in graphs. Significant differences were considered: n.s., nonsignificant difference; *P <* *0.05; **P <* *0.01; and ***P <* *0.001 by unpaired Student's *t*‐test.

## Results

### Apc^min716/+^ mice spontaneously develop colon tumors and splenomegaly

**Figure 1:**
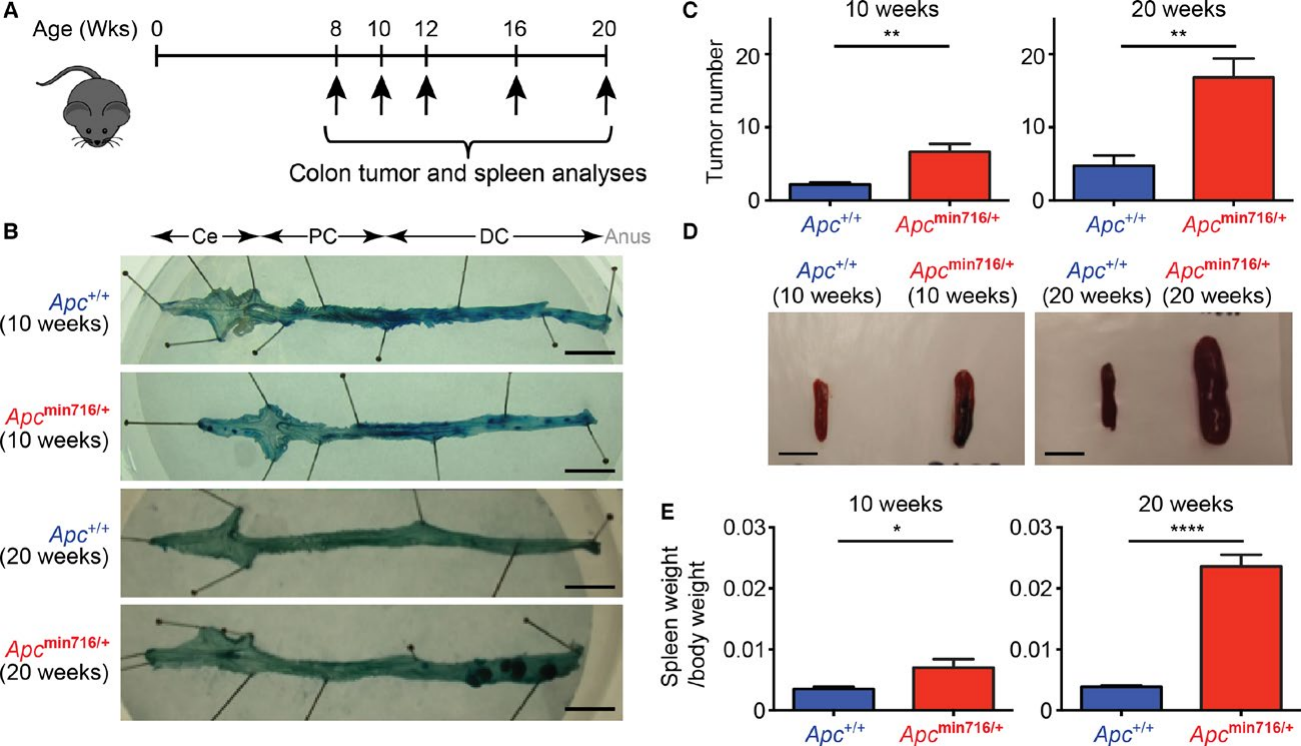
*Apc* ^min716/+^ mice develop colon tumors and splenomegaly over time. (A) A schematic of the experimental timeline for observing colon tumors and spleen sizes in mice. (B) The colons of 10‐week‐ and 20‐week‐old wild‐type (*Apc* ^+/+^) and *Apc* ^min716/+^ mice were resected, stained with methylene blue, and tumors were counted using a dissecting microscope. Ce, cecum; PC, proximal colon; and DC, distal colon. Scale bars, 1 cm. (C) Average tumor numbers from 10‐week‐old *Apc* ^+/+^ mice (*n* = 6) and *Apc* ^min716/+^ mice (*n* = 6), or 20‐week‐old *Apc* ^+/+^ mice (*n* = 4) and *Apc* ^min716/+^ mice (*n* = 5) were quantified. (D) The spleens of the 10‐week‐ and 20‐week‐old *Apc* ^+/+^ and *Apc* ^min716/+^ mice were resected and imaged. Scale bars, 1 cm. (E) Spleen weight, normalized to body weight, from 10‐week‐old *Apc* ^+/+^ mice (*n* = 6) and *Apc* ^min716/+^ mice (*n* = 6), or 20‐week‐old *Apc* ^+/+^ mice (*n* = 4) and *Apc* ^min716/+^ mice (*n* = 5) were quantified. *P <* *0.05; **P <* *0.01; ****P <* *0.0001 by Student's *t*‐tests.

As reported previously 33, *Apc* ^min716/+^ mice develop a large number of adenomas in the colon and exhibit a reduced life span (~6 months), thus modeling human colon cancer to certain extents. In order to examine the tumor development present within the colon, *Apc* ^min716/+^ mice and their wild‐type (*Apc* ^+/+^) littermates were aged according to the schematic in Figure 1A. As conveyed by whole mount methylene blue staining of the colon for aberrant crypt foci that is indicative of neoplastic areas with hyper‐proliferative densely packed cells, *Apc* ^min716/+^ mice spontaneously developed adenomas between 10 and 20 weeks of age, with majority of adenomas present within the final 2 cm of the distal colon, some in the cecum, and occasionally some within the proximal colon (Fig. 1B–C). In contrast, relatively few aberrant crypt foci were present within the colons of wild‐type *Apc* ^+/+^ mice (Fig. 1B–C).

It was previously reported that the spleens were enlarged in tumor‐laden *Apc* ^Min/+^ mice 17, 18, 19. Similarly, we observed splenomegaly in *Apc* ^min716/+^ mice, dramatically increasing with age as demonstrated when spleens were imaged alongside age‐matched *Apc* ^+/+^ controls (Fig. 1D). There were minor differences in the size of the spleens derived from *Apc* ^min716/+^ and *Apc* ^+/+^ mice at 10 weeks old; however, by 20 weeks of age, the spleens of the *Apc* ^min716/+^ mice increased significantly (Fig. 1E). Of note, there is no statistically significant difference in animal size when examining age‐match *Apc* ^+/+^ and *Apc* ^min716/+^ mice (data not shown), which suggests that the differences in spleen size are not caused by an increase in body weight. Instead, the increases in spleen size and tumor burden in aged *Apc* ^min716/+^ mice may provide additional morphological and pathological markers to evaluate colon tumor development.

### Splenomegaly is positively correlated with tumor development in Apc^min716/+^ mice

**Figure 2:**
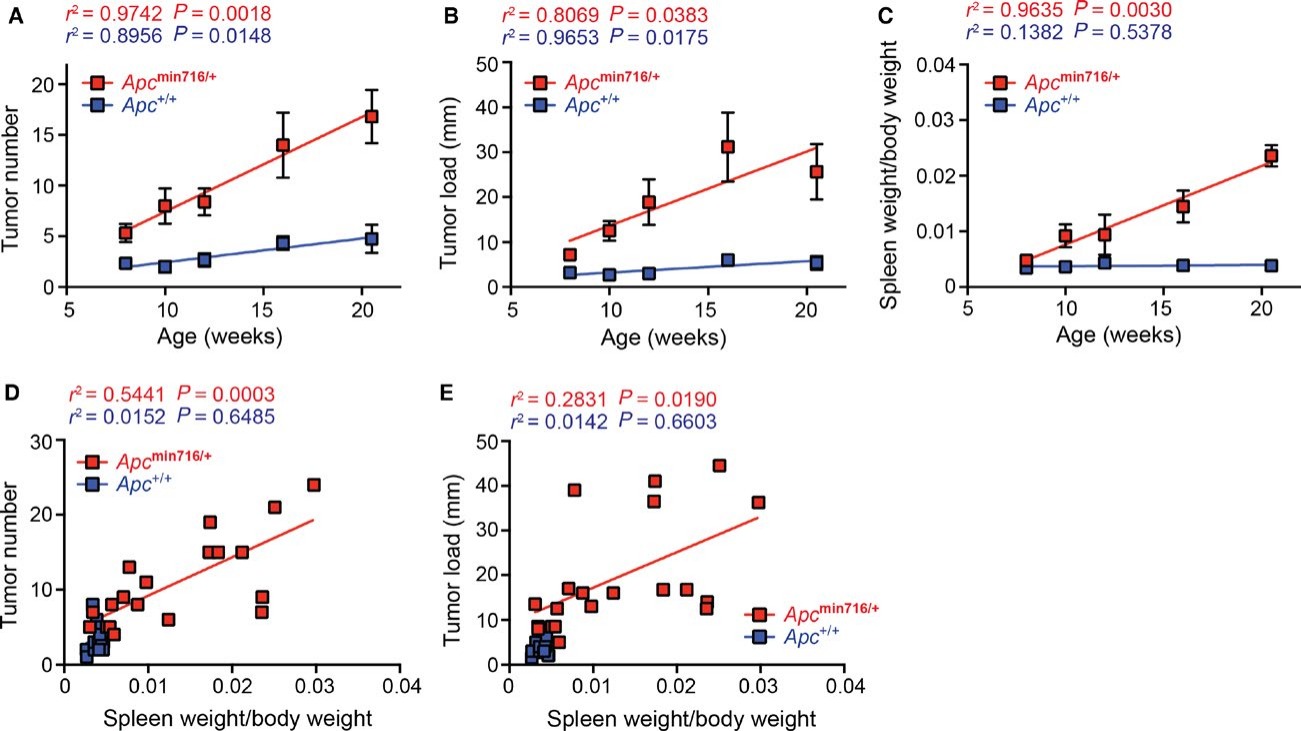
Colon tumors and splenomegaly increase with age and are positively correlated in *Apc* ^min716/+^ mice. The colons and spleens were derived from *Apc* ^+/+^ and *Apc* ^min716/+^ mice at the indicated ages and the colons were stained with methylene blue to determine the number and size of colon tumors. (A–C) The tumor number (A), tumor load (B), and spleen weight, normalized to body weight (C) from indicated mice (3–5 mice per group per time point) were plotted against the age. (D–E) Linear regression analysis of the tumor number (D) and tumor load (E) versus normalized spleen weight in *Apc* ^+/+^ and *Apc* ^min716/+^ mice.

Previous studies reported that splenomegaly was observed in the tumor‐laden *Apc* ^Min/+^ mice 17, 18, 19; however, the connection between spleen size and tumor progression has not been established. The dramatic changes in tumor burden and spleen size in 20‐week‐old *Apc* ^min716/+^ mice versus 10‐week‐old ones (Fig. 1B–C) led us to speculate that the increase in spleen size could reflect the tumor development in aged *Apc* ^min716/+^ mice. We therefore sought to explore the spleen size‐tumor development correlation in *Apc* ^min716/+^ mice with varying age. As expected, colon adenoma development, as conveyed by tumor number and tumor load, was positively correlated with the age of *Apc* ^+/+^ and *Apc* ^min716/+^ mice, albeit the magnitude is far greater for the *Apc* ^min716/+^ mice (Fig. 2A–B). Interestingly, in line with the spleen size illustrated in the Figure 1C, the spleen weight, normalized to body weight, increased over time in *Apc* ^min716/+^ mice, whereas the normalized spleen weight in *Apc* ^+/+^ mice did not change with age (Fig. 2C). Moreover, the increase in splenomegaly is positively correlated with the age of the *Apc* ^min716/+^ mice, and the divergence in spleen size between *Apc* ^+/+^ and *Apc* ^min716/+^ mice started as early as 10 weeks old and became most obvious at the 20‐week time interval (Fig. 2C). The positive correlations between colon tumor development versus mouse age and normalized spleen size versus mouse age strongly suggest that the splenomegaly process could coincide with the tumorigenesis in the *Apc* ^min716/+^ mice. Indeed, the number of tumors remained positively correlated with the normalized spleen weight in the *Apc* ^min716/+^ mice regardless of age (Fig. 2D). There was no significant correlation between the tumor number and normalized spleen weight in *Apc* ^+/+^ controls (Fig. 2D), which is unsurprising given the low abundance of hyperplastic foci staining and the lack of change in spleen size over time in the *Apc* ^+/+^ animals (Fig. 2A and C). Likewise, colon tumor load followed the same trend as tumor number, which increased and positively correlated with the normalized spleen weight in *Apc* ^min716/+^ mice (Fig. 2E). These data demonstrate that spleen size, as measured by normalized weight, is predictive of colon tumor development in *Apc* ^min716/+^ mice.

### In vivo ultrasound imaging reliably reveals spleen size and tumor development in Apc^min716/+^ mice

**Figure 3:**
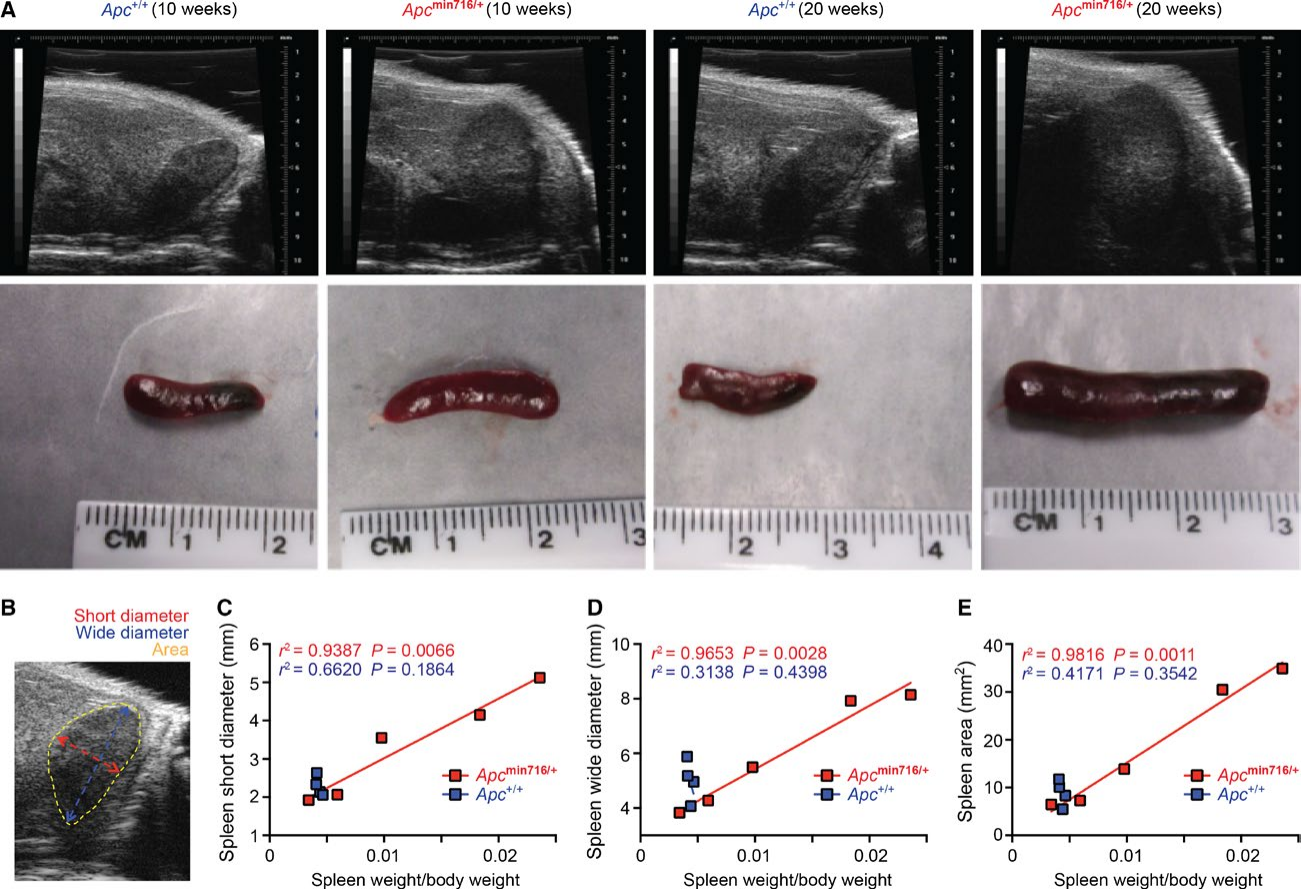
Dimensions of spleens from ultrasound images are correlated with spleen weight. (A) Ultrasound cross‐sections of spleens were imaged in vivo from 10‐week‐ and 20‐week‐old *Apc* ^+/+^ and *Apc* ^min716/+^ mice, upper panels. After ultrasound imaging was completed, mice were killed and the spleens were resected and imaged, bottom panels. (B) A schematic of the measurements of short diameter, wide diameter, and area of the spleen taken from the ultrasound images. (C–E) Linear regression analysis of the spleen short diameter (C), wide diameter (D), and spleen area (E) versus the normalized spleen weight in *Apc* ^+/+^ (*n* = 4) and *Apc* ^min716/+^ (*n* = 5) mice.

The established positive correlation between spleen size and colon tumor number and tumor load in *Apc* ^min716/+^ mice with varying ages (Fig. 2D–E) suggests that assessing spleen size could serve as an alternative in vivo approach to systemically monitor tumor development in *Apc* ^min716/+^ mice. We employed ultrasound imaging, a quick and noninvasive technique that provides a cross‐sectional image of a variety of organs including the spleen without requiring the killing of the animal 28, 29, 30, 31. As illustrated by ultrasound images taken from the abdomen of anesthetized animals, the difference in spleen size was noticeable and immediately visible in the sonograms of the 10‐ and 20‐week‐old *Apc* ^min716/+^ and *Apc* ^+/+^ mice, respectively (Fig. 3A). To ascertain that spleen sonograms could be used accurately to estimate the physical spleen sizes in vivo, the spleens were resected from the killed mice, after the sonograms were taken, for spleen size and weight measurements. Our results demonstrate that the sonogram image appropriately reflects the size of the spleen derived from each mouse (Fig. 3A), which is further supported by our analyses of spleen sonograms and normalized spleen weight. With the ultrasound machine positioned over the widest region of the spleen, we analyzed the diameters of the shortest part, the widest part, and the area of the spleen from the sonogram images (Fig. 3B). When the dimensions measured from the ultrasound images were plotted against the body weight‐normalized spleen weight, we observed strong positive correlation between the short diameter, wide diameter, and area of the spleen and the physical spleen weight (Fig. 3C–E), supporting the use of the ultrasound imaging to reliably measure the spleen size in mice in vivo. Moreover, consistent with the linear correlation between the normalized spleen weight and colon tumor number and load (Fig. 2D–E), the dimensions measured from the spleen ultrasound images were also positively correlated with colon tumor development, with both tumor number and tumor load, in the killed *Apc* ^min716/+^ and *Apc* ^+/+^ mice after sonograms were completed (data not shown). Hence, imaging the spleen size in vivo by ultrasound may serve as a unique tool to monitor and estimate the colon tumor development in *Apc* ^min716/+^ mice.

## Discussion

Splenomegaly has been recognized in the tumor‐laden *Apc* ^Min/+^ mice 17, 18, 19; however, the etiology of splenomegaly and the relationship between spleen enlargement and tumor formation and progress are not fully understood. Here, we reveal a positive correlation between the degree of colon tumor development and the extent of the splenomegaly in *Apc* ^min716/+^ mouse model of colon cancer. As expected, the number and load of colon tumors spontaneously increases in *Apc* ^min716/+^ mice over time, which most likely facilitates the accumulation of further mutations and the complete loss of *Apc* gene activity. Interestingly, in parallel to the increased number and load of colon tumors, the dimensions and weight of the spleen increased in *Apc* ^min716/+^ mice of varying ages, which is unrelated to the animals' body weight growth over time. The divergence between the spleen size from *Apc* ^+/+^ and *Apc* ^min716/+^ mice is exhibited more profoundly in older animals where colon tumors are fully developed. The significance of the correlation between splenomegaly and tumor development in *Apc* ^min716/+^ mice across ages provides the rationale for measuring spleen size as a novel approach to monitor colon tumor development in vivo.

Cancer development is believed to be a chronic and gradual process, with the formation and progression of tumors needing substantial amount of time. Hence, monitoring tumor development in cancer animal models requires striking a careful balance between allowing enough time for tumor growth and capturing the best time windows to reach biological significance. This makes the study of internal cancers particularly challenging without access to methods to observe tumor progression directly. The advancement in imaging techniques and tools has bettered our understanding of the molecular events surrounding tumor formation and progression 4, 20, 22, 24, 25, 26. For instance, endoscopy and colonoscopy procedures have been widely used to image and monitor colon tumor development in vivo in both clinical application and preclinical studies 36, 37. However, these procedures will not allow imaging of the entire intestinal tract beyond the local tumor development in the distal colon, thus reflecting local, rather than systemic, tumorigenesis. Moreover, the cost and expertise required for the endoscopy and colonoscopy procedures prevent their widespread use. Here, our study suggests that ultrasound imaging of the spleen could be used as a proxy to monitor the general level of tumor formation within the animal. It is noteworthy that ultrasound imaging of the spleen, as a noninvasive, can be done repeatedly without detrimental effects on the animal and images can be used to determine the growth of the spleen, which is positively correlated with tumor development over time. The ease and time required to take ultrasound images of experimental mice is minimal compared to the time invested in repeating experiments when animals were killed at suboptimal time windows to observe differences in tumor formation. Although this technique does not provide a detailed record of the internal microenvironment, it could serve as an indicator of the general tumor environment within individual mice. Ultrasound imaging is already an extensively used and well‐established technique in many research institutes that can be co‐opted for determining the progression of colon cancer in mouse models of the disease.

## Conflict of Interest

None declared.
